# Raw and Cooked Quality of Gilthead Seabream Fillets (*Sparus aurata*, L.) after Mild Processing via Osmotic Dehydration for Shelf Life Extension

**DOI:** 10.3390/foods11142017

**Published:** 2022-07-07

**Authors:** Niki Alexi, Konstantina Sfyra, Eugenia Basdeki, Evmorfia Athanasopoulou, Aikaterini Spanou, Marios Chryssolouris, Theofania Tsironi

**Affiliations:** 1 Food Quality Perception and Society Science Team, iSENSE Lab, Department of Food Science, Faculty of Technical Sciences, Aarhus University, Agro Food Park 48, 8200 Aarhus N, Denmark; ksfyra@food.au.dk; 2 Laboratory of Food Process Engineering, Department of Food Science and Human Nutrition, Agricultural University of Athens, Iera Odos 75, 11855 Athens, Greece; eugeniaba97@gmail.com (E.B.); efiatha@aua.gr (E.A.); sp.katerin@aua.gr (A.S.); ftsironi@aua.gr (T.T.); 3 SuSea BV, High Tech Campus 1, 5656 AE Eindhoven, The Netherlands; info@susea.co

**Keywords:** quality index method, microbial spoilage, tetrad discrimination testing, sensory descriptive analysis, shelf life, freshness, minimal processing, seafood preservation

## Abstract

The current study aimed to explore the effects of mild processing for shelf-life extension on the raw an-d cooked quality of gilthead seabream fillets stored at 2 °C. Control and Treated (via osmotic dehydration) fillets were sampled at the beginning (D1), middle (D5) and end (D7) of commercial shelf life. The raw quality was evaluated via the quality index method (QIM), microbial measurements and for D1 through tetrad discrimination testing. The cooked quality was evaluated for the same samples via sensory descriptive analyses with a trained panel. The tetrad results indicated similar characteristics between treatments for raw fillets on D1 and a 29% shelf-life extension for Treated fillets vs. the Control ones, defined by Quality Index Method and microbial measurements. The raw quality was reflected in the cooked quality of the tissue, with the Treated fillets exhibiting less intense spoilage-related sensory attributes as well as enhanced or retained freshness-related attributes throughout storage, when compared to the Control ones. A range of treatment induced sensory characteristics, partly associated to Maillard reactions, were developed in the Treated fillets. Overall, the treatment affected positively both the raw and cooked quality of the fillet, showing promising results as a shelf-life extension method for fish fillet preservation.

## 1. Introduction

Animal derived products, such as fresh meat and fish products, are amongst the most resource demanding yet perishable food product categories. Examining fish products specifically, 35% of the fisheries and aquaculture production is wasted globally. This underlines the importance of novel tools and processes that can assist in minimizing these losses and thereby reduce the pressure and enhance the sustainability of the current production systems [1].

When examining the losses at a retail level, limited product shelf life can be a major contributor [2]. This is especially applicable to fresh fish fillets, a preferred product choice in many countries, yet with a limited shelf life [3,4,5]. To address this, a range of processing and preservation methods, such as salting, curing, drying, marinating, canning, smoking etc., have been developed and applied traditionally post-harvest to extend the shelf life of different fish products [6,7]. Other common approaches applied to extend the shelf life of fish is refrigeration and freezing after harvesting, which delays or inhibits microbial growth and physicochemical reactions, resulting in quality deterioration during storage.

Besides the processing effects on shelf life, the application of different technologies can alter the sensory and nutritional characteristics of the product, affecting its organoleptic quality (appearance, aroma, taste-flavor, texture) and having in some cases detrimental effects on its nutritional value [7,8]. Due to the importance of the appearance of the product in retail (choice) and its flavor and texture during consumption (eating experience), treatment effects on the sensory aspects of both raw and cooked fish products are important to consider, besides the nutritional alterations and shelf life extension achieved via processing [9,10]. 

Moreover, whereas processing can result in high sensory and nutritional quality, the perceived freshness is an established part of fish product quality [7,9,10]. This is shown by the higher perceived quality associations of consumers to fresh versus processed fish products and a preference for food products that retain their natural appearance [5,11]. Indeed, nowadays the consumer demands fresh or minimally processed foods of high quality and nutritional value, natural and safe, with the minimum addition of preservatives [12]. Within these frames, preservation and processing methods that increase the shelf life of fish products, while not compromising on the quality of sensory characteristics, are sought by the industry [13].

Considering the role of water activity (*a_w_)* in microbial growth and thereby perceived freshness, the current work examined a minimal processing method consisting of a mild tissue dehydration (ranging 5–10%, corresponding to final a_w_ values between 0.93–0.95), for its ability to extend shelf life, while not affecting the raw quality of gilthead seabream (*Sparus aurata* Linnaeus, 1758) fillets [14,15,16]. Osmotic dehydration has been reported as an effective non-thermal processing method, for delaying growth of spoilage bacteria and extending the shelf life of fresh fish. Several osmotic media have been investigated, such as sucrose, maltodextrin, trehalose and glycerol at different processing times and temperatures [16,17,18]. Towards the current trend for fresh-like food products with improved quality and extended shelf life, the systematic evaluation of consumer perception for minimally processed fish is a prerequisite. 

In the present study, the shelf life extension was benchmarked by the Quality Index Method (QIM) and supported by microbial analyses of the tissue [19]. To ensure that the sensory characteristics of the raw product were not altered, sensory discrimination testing was performed [20]. Moreover, due to the importance of the eating quality and the sensory characteristics upon consumers’ acceptance (and re-purchase) the treatment was evaluated regarding its effects on cooked tissue throughout its commercial shelf life via generic descriptive analyses (DA) [20]. Gilthead seabream was chosen as a species of importance due to its production numbers and contribution to the growing aquaculture sector [21]. Gilthead seabream (*Sparus aurata*) is one of the most cultured species in the Mediterranean area, with a total production of almost 200,000 tons in 2019 [22]. Besides the desired organoleptic characteristics (aroma, taste) of the species, gilthead seabream has a high nutritional value, reflected in flesh which contains a high concentration of proteins, essential amino acids and polyunsaturated fatty acids [23,24]. Although usually sold as whole fish, gilthead seabream filleted products have high commercial potential but suffer from short shelf life [16,25,26].

The specific aims of the current work were to evaluate:

- the shelf-life extension achieved by the treatment and raw quality of the Treated gilthead seabream fillets versus the Control ones via sensory (QIM, Tetrad discrimination) and microbial methodologies
- the cooked quality and the sensory profile of the Treated gilthead seabream fillets versus the Control ones throughout shelf life via sensory descriptive analysis (DA).

## 2. Materials and Methods

### 2.1. Fish Samples, Processing and Storage Conditions

The fresh gilthead seabream (*Sparus aurata*, Linnaeus, 1758) fillets used for the pre-trials and the main experiment were commercially obtained from Select Fish SA, Athens, Greece. Fish destined for the main experiment were harvested on 1 March 2022. Pre-processing of fish into fillets was conducted on the following day at the Select Fish SA facilities. The weight of the obtained fish fillets ranged from 90–120 g. Directly after pre-processing 400 fillets were divided into seven polystyrene boxes (~6 kg each), with the addition of an appropriate amount of flaked ice. Fillets were coated with high density polyethylene films to avoid direct contact of fish flesh with ice or fish skin. After packaging, the boxes were transported directly to the Agricultural University of Athens (AUA), Department of Food Science and Human nutrition, Laboratory of Food Process Engineering. Upon arrival, the ice was removed from the polystyrene boxes, which were then placed in refrigerated storage at 2 °C (±0.2). The packaging date and expiry date set on the package were the second and ninth of March 2022, respectively. 

The experimental trials at AUA were initiated the following day with the processing of the fillets, which was assigned as Day 0 (D0) of the experiments. For this purpose, fish fillets were divided randomly into two groups. Half of the fillets remained untreated at refrigerated storage (Control fillets). The other half of the fillets were mildly processed via osmotic dehydration (Treated-SUSEA fillets). The Osmotic dehydration was applied on fish fillets using a hypertonic aquatic solution optimized by SuSea BV (SuSea BV, Eindhoven, The Netherlands). Fish was processed in an aquatic solution consisting of 30% glycerol, 10% dextrose and 5% NaCl for 40 min at 15 °C [27,28]. Directly after processing, the fillets were dried onto filter paper and placed back in the polystyrene boxes for storage at 2 °C.

### 2.2. Design of Freshness and Shelf Life Experiments of Raw Products

The shelf-life trials of the raw fillets were initiated on D1 of the experiments with subsequent analyses and sampling being conducted on D1, D5, D7, D8, D11 and D13. This included up to D11 for both the Control and Treated fillets, whereas at D13 only the Treated fillets were evaluated due to the expiry of the Control ones. The storage time and sampling frequency were defined according to the Control fillets’ expected shelf life (expiry date) in order to (i) capture three freshness stages (High: D1; Medium: D5; Low: D7), (ii) monitor the transition of the fillets from acceptable to unacceptable for consumption (D7–D8) and (iii) capture the expected shelf-life extension (up to 50%) for the Treated fillets. 

The freshness and corresponding shelf life of the gilthead seabream fillets undergoing storage at 2 °C were assessed by the Quality Index Method (QIM) and through microbial evaluations. Five fillet replicates were used per treatment at each shelf-life date examined. In addition to the shelf life trials, a Tetrad test (ISO 6658:2017) with 36 assessors was conducted on D1 to determine whether any perceivable differences between the raw fillets of the two treatments existed. 

#### 2.2.1. Discrimination Testing (Tetrad)

Discrimination testing was conducted at D1 of storage, following the Tetrad method. For this purpose, N = 36 assessors with no previous experience in sensory science were recruited. Assessors were presented with four fillets (two of each treatment) on 3-digit coded paper plates that did not reflect light, and they were asked to group the samples into two pairs taking into account their appearance, odor and texture by hand. The Tetrad evaluation was conducted at room temperature on tablets using the 2020 EyeQuestion^®^ Software (Version: 5.2.3, Logic8. BV, Elst, The Netherlands) and followed a fully randomised sample presentation among panellists. 

The statistical power of the test and the data analysis were calculated according to the V power program written by Virginie Jesionka and based on the Discrimination Test Planning and Analysis Tools developed by Tom Carr (Carr Consulting, 1215 Washington Ave, Suite 203, Wilmette, IL 60091, USA). Due to the natural high variability of the within batch variation of the samples, the following parameters were taken into account when setting up the test: d’ = 1.2, a = 0.1 and Power = 0.9. As a result, the minimum number of assessors required to participate in the test was 32 and our actual sample size was N = 36. For the data analysis and the setting of parameters before the test, the Thurstonian model for a Similarity test was followed.

#### 2.2.2. Quality Index Method (QIM)

##### Development and Validation of the QIM Scheme for Gilthead Seabream Fillets

The QIM scheme development was based on the methodology described by Martinsdóttir, Sveinsdottir, Luten, Schelvis-Smit and Hyldig [19]. Previous work describing QIM schemes and sensory changes of whole fish and fillets of fresh gilthead seabream was used for its development and parameter definition [29,30,31]. This scheme was then validated through pre-trials by examining and recording the changes occurring at fresh gilthead seabream fillets stored at 2 °C for the defined appearance, odor and texture parameters until spoilage. During the pre-trials, fillets corresponding to different freshness stages were stored (−40 °C, storage duration: 1–2 weeks) in order to create reference material for the introductory QIM evaluation session.

The validated scheme included six appearance, odor and texture parameters (Table 1). For each of the parameters a demerit point scale of up to 3 points, accompanied by appropriate descriptions reflecting the quality changes with storage, was used. Zero (0) corresponded to very fresh gilthead seabream fillet, whereas the demerit points increased according to the spoilage degree of the fillet with two (2) and three (3) points corresponding to spoiled fish appearance/texture and odor parameters, respectively. The Quality Index (QI) of the scheme ranged from 0 to 13 points.

##### QIM Evaluation

A panel consisting of eight (8) judges was employed for the QIM. The panel participated in an initial training session during which the panel leader introduced the process, preliminary QIM scheme and point scale used to evaluate each parameter, by presenting reference samples corresponding to different freshness stages stored during the pre-trials. 

Five to eight judges participated at each of the QIM freshness evaluation sessions conducted on D1, D5, D7, D8, D11 and D13. The fillets of each treatment were presented to the panelists in triplicates on 3-digit coded white paper plates that did not reflect light at room temperature. The QIM scheme evaluation was performed on tablets using the Compusense20 software (Version 22, Compusense Inc., Guelph, ON, Canada). For each of the fillets the judge had to evaluate the parameters using the QIM demerit point scheme and at the end indicate whether the fillet was acceptable for consumption. A panel discussion was conducted after the initial evaluation session to ensure panel consensus on the definition of the individual parameters and scale points. 

The QIM results of the Control and Treated fillets are presented as the total QI (sum of demerit points), averaged across assessor and replica, per storage day of the freshness trials. The fitted linear model equation and R^2^ of the fit were calculated in Microsoft Excel 2016.

##### Microbial Evaluation

For microbiological enumeration, a representative sample (10 g) was collected from fish fillets (including skin and flesh), transferred to a sterile stomacher bag with 90 mL sterilized Ringer solution (Merck, Darmstadt, Germany) and homogenized for 60 s with a Stomacher (BagMixer^®^, Interscience, Saint-Nom-la-Breteche, France). Zero point one milliliters of tenfold serial dilutions of fish homogenates were spread on the surface of appropriate media in Petri dishes for microbial enumeration. Total aerobic viable count (TVC) was enumerated on Plate Count Agar (PCA; Neogen, Lansing, MI, USA) after incubation at 25 °C for 72 h. *Pseudomonas* spp. were enumerated on Cetrimide Agar (CFC; Condalab, Torrejon De Ardoz, Spain) after incubation at 25 °C for 48 h.

Two replicates of at least three appropriate dilutions were enumerated. The microbial growth was modelled using the Baranyi growth model (Baranyi and Roberts, 1995). For curve fitting the program DMFit (IFR, Institute of Food Research, Reading, UK) was used (available at http://www.combase.cc/index.php/en/, accessed at 1 June 2022). Kinetic parameters of microbial growth such as the rate (k), lag phase (λ) and final population levels (N_max_) were estimated. Furthermore, the linear correlations between different QI and microbial measurements across shelf-life sampling dates were studied by calculating simple linear regression models in Microsoft Excel software, 2016. The relationships were evaluated by an F-test with a significance level of 5%, which are included as correlation coefficients (r) accompanied with their respective *p*-values (*p*_r_).

### 2.3. Sensory Evaluation of Cooked Products

#### 2.3.1. Samples Storage, Preparation, Cooking Trials and Presentation 

Control and Treated fillets destined for sensory evaluation via generic Descriptive Analysis (DA) from D1, D5 and D7 were vacuum packed and stored at −40 °C at the AUA (Athens, GR), Department of Food Science and Human Nutrition, Laboratory of Food Process Engineering. The samples were then transported frozen to Aarhus University, Department of Food Science (AU FOOD, Aarhus, DK) where they were stored at −20 °C, until analysis. The thawing process involved placing the vacuum-packed fillets in a thermal chamber (Termaks, series 6000) overnight at 1 ± 0.1 °C to allow for gradual defrosting of the tissue. The time of frozen storage from sampling at the AUA facilities till thawing of tissue for sensory analysis at the AU facilities did not exceed a month.

Prior to the initiation of the sensory evaluation process, cooking trials were performed. The sample preparation for the cooking trials was conducted in like manner as for the sensory evaluation. This involved removing the dorsal line and ventral part of the fillet, due to the their higher fat content and contact with the internal organs, which can result in deviations in the volatile composition (and thereby sensory profile) [32]. Thereafter, the dorsal part of the fillet remaining was cut into approximately 4 cm × 3 cm rectangles (15–20 g), and placed in ceramic containers that were covered by aluminum foil [33]. For the culinary preparation of the samples, a series of different time (7–10 min) and temperature (80–100 °C) combinations were examined. The final cooking temperature was set at 100 °C for 7 min (relative humidity of the oven chamber was kept at 60%) and was performed in iCombi^®^ Pro RATIONAL ovens. The cooking process as well as temperature (60 °C) and resting time (maximum 10 min) of samples in the thermal chamber were validated with the sensory panel, to avoid drying of the tissue. 

Six samples were profiled by the DA method corresponding to the Control fillets from D1 (CD1), D5 (CD5) and D7 (CD7) and Treated SUSEA fillets from D1 (SD1), D5 (SD5) and D7 (SD7). 

#### 2.3.2. Descriptive Analysis and Profiling of Cooked Products

All sensory training and evaluation sessions were conducted at the AU-FOOD iSense lab facilities (adhering to ISO standards ISO 8589:2007). A screened and trained panel (according to ISO 8586:2012) consisting of 10 assessors (5 male and 5 female, age: 23–48) with previous experience in the sensory evaluation of food and fish products in specific was employed. Due to COVID protocols the panel was divided during the whole process in morning and afternoon shifts to adhere to social distancing protocols established at AU-FOOD. 

The DA process was conducted within two weeks and occupied a total of seven days. This included: (i) three 2 h generic training sessions placed on different days, (ii) one vocabulary generation and two training sessions with the Control and Treated samples accounting to a total of 5 h, placed on three separate days and (iii) three evaluation sessions accounting to 3 h and placed on two days. 

The three generic training sessions were dedicated to training and reference development for attributes found in fish products. A variety of different products were presented to the panel as references, including fish fillets, seafood products and algae products as well as other relevant references (e.g., boiled potatoes, mushrooms, lactic acid). The aim of these sessions was to clarify the vocabulary and achieve alignment and consensus amongst the panel. 

The vocabulary session involved the tasting of extreme samples (CD1, SD1, CD7 and SD7). During this session, assessors were asked to generate terms describing all the characteristics of appearance, odor, taste, flavor, texture, aftertaste and mouthfeel (after swallowing) perceived in the samples. This resulted to an initial vocabulary list containing 48 attributes. The remaining training involved three 1 h sessions: (a) sample pair comparisons, (b) booth sample training, including two blind duplicates to evaluate repeatability, and (c) sample pair comparisons. For booth sample training all generated attributes in the initial vocabulary list were included and evaluated for their intensity on a 150 mm scale, anchored at 0 and 150 mm with “None” to “Very high”, respectively (Exceptions for specific attributes can be found in Table 2). The booth training was conducted on tablets using the Compusense20 software. After this process the initial attribute list was reduced to 37 attributes, which were used in the final DA evaluation and can be found along with their definitions and references in Table 2. Panel Check V1.4.2 was used to provide panel feedback for improving the assessors’ performance throughout the training process. Prior to the initiation of the DA evaluation sessions, specific references were presented to the assessors to ensure clarification of unclear attributes.

The DA evaluation was conducted at the AU-FOOD iSense lab sensory booths in three sessions over two days. Data were registered on tablets using the Compusense20 software. The assessors evaluated three replications of each sample following a block randomised design (one replication per session). Samples were kept in a thermal chamber (60 °C) and time between culinary preparation and serving did not exceed 10 min. They were served in aluminum foil-covered white ceramic containers, blind coded with 3-digit numbers, and in randomized (William Squared Design) monadic order, accounting for first order and carry over effects [34]. The attributes line scale and anchors used were the same as in booth training described above. Modalities were evaluated in the following order: odor for which assessors were instructed to lift the aluminum (and place it back after each sniffing), appearance, taste, flavor, texture and after-taste/-mouthfeel. Aftertaste and mouthfeel were evaluated 20 sec after sample swallowing and a break of 1 min was included in-between samples. A combination of two palate cleansers (green apple, cucumber, sparkling water and crackers) were used and assessors had access to tap water. 

#### 2.3.3. Statistical Analyses

A 2-way ANOVA model with interaction (factors: treatment, day, treatment × day) was performed to identify significant (*p* < 0.05) effects of the design factors on the perception of individual attributes. A sequential elimination of insignificant (*p* < 0.1) interaction effects was performed. Fisher’s LSD was used as a post-hoc test to identify sample groupings according to the design factors. Attributes showing a tendency of significance were considered in combination with the results of the post-hoc test, due to the inherent inter fillet variability.

Principal Component Analysis (PCA) was furthermore employed for the visualization of the sensory maps. Two PCA (co-variance) analyses were conducted; one including the design factors of the experiment as observations and the samples as supplementary observations, and one including only the samples as observations. In all cases, only attributes that varied significantly or showed a tendency of variation (*p* < 0.1) according to the 2-way factorial ANOVA model were included in the calculation of the PCA models as variables. The PCA bi-plot (after Varimax rotation) with the design factors of the experiment as observations is included in the main body of the results; the PCA bi-plot with the samples as observations is included in the Supplementary material. In the latter, Convex hulls were calculated for the samples according to the partial bootstrap method [35] based on the most extreme observations. 

Agglomerative Hierarchical Clustering (AHC) was furthermore used to classify the samples according to their similarity and dissimilarity based on the raw sensory data. AHC on similarity was calculated using Pearson’s correlation coefficient, as agglomeration method, complete linkage and as truncation entropy. AHC on dissimilarity was calculated using Euclidian distance, as agglomeration method, Wards method and as truncation entropy.

All statistical analyses (ANOVA, PCA, AHC) were performed in XLSTAT^®^ software, 2016 (Addinsoft™, New York, NY, USA), whereas Panel Check V1.4.2 was used to assess the assessor performance and data obtained from the DA evaluation prior to the initiation of statistical analyses.

## 3. Results

### 3.1. Freshness and Shelf Life of Raw Fillets

The a_w_ of fish was 0.99 and 0.95 for the untreated and the treated samples, respectively. The respective values for flesh moisture were 2.4 and 1.9 g water/g dry mass. Solid gain after treatment was 0.16 g total solids/g initial dry mass. Salt content in fish flesh was 0.4 and 1.1% before and after treatment, respectively. 

Out of 36 assessors who performed the Tetrad test, 15 could identify the “correct” pairs. Given the setup parameters of the analysis (included in the respective 0 section), this was translated to a significant similarity amongst the raw Control and Treated fillets with a confidence of 87%, whereas *p*-value of difference was calculated at 0.187. 

**Figure 1:**
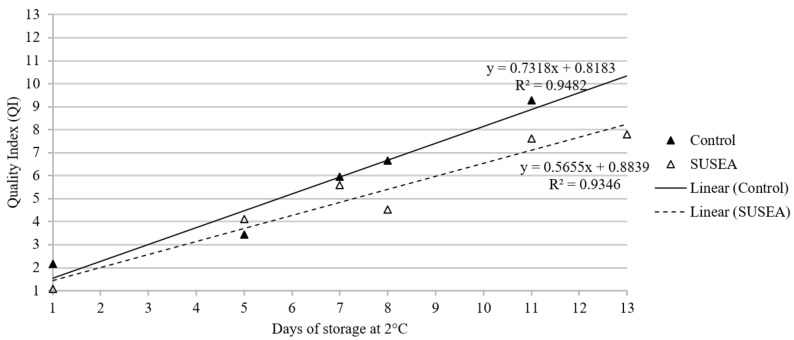
Quality index of gilthead seabream fillets stored isothermally at 2 °C for the Control (untreated) and SUSEA (Treated) fillets. Continuous lines represent the statistical fit of a linear model. The R^2^ and equation of the linear model fit are included in the plot (Top: Control; Bottom: SUSEA).

The QI for seabream fillets ranged from 0–13 demerit points and increased linearly with storage time for both the Control and Treated with R^2^ of 0.95 and 0.93, respectively (Figure 1). The QI progression with storage varied between the Control and Treated fillets showing a clear separation on D8 of storage and onwards, with the Treated fillets being evaluated as having consistently a lower QI than the Control ones. The rejection QI (lowest QI for which a fillet was assessed as unacceptable for consumption from at least one of the judges) was determined at six (6) demerit points for the gilthead seabream fillets. According to the QI linear model equations of the untreated (y = 0.7318x + 0.8183) and processed (y = 0.5655x + 0.8839) fillets (Figure 1), this corresponded to an expiry on D7 and D9 of the experimental trials for the Control and Treated batches, respectively. Within these frames, the extension of shelf life for the Treated fillets was calculated at 29%, when compared to the Control ones. 

**Figure 2:**
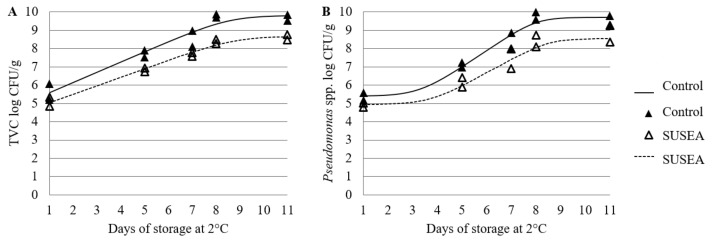
Microbial growth models of (**A**) Total Viable Counts (TVC) and (**B**) *Pseudomonas* spp. in gilthead seabream fillets stored isothermally at 2 °C for the Control (untreated) and SUSEA (Treated) fillets. Continuous lines represent the statistical fit of the Baranyi model.

The initial microbial load of the Control fillets was 5.6 ± 0.6 and 5.4 ± 0.3 log CFU/g for TVC and *Pseudomonas* spp., respectively, while the corresponding values for the Treated ones were 5.1 ± 0.4 and 4.9 ± 0.2 log CFU/g. Growth curves of TVC and *Pseudomonas* spp. in gilthead seabream fillets stored isothermally at 2 °C were fitted to the Baranyi Growth Model (Figure 2A,B) and the growth kinetic parameters were determined (R^2^ > 0.9 in all tested cases). No lag phase was observed for TVC for either of the processing conditions, whereas for *Pseudomonas* a lag phase of 2.3 and 2.7 days was observed for the Control and Treated fillets, respectively. Microbial growth rates were lower in the Treated when compared to the Control fillets (0.559 and 0.464 for TVC, and 0.908 and 0.742 for *Pseudomonas* spp. in Control and Treated, respectively). *Pseudomonas* spp. dominated spoilage in all processing conditions, with final microbial populations ranging 8.9–9.8 log CFU/g and 8.7–9.8 log CFU/g for TVC and *Pseudomonas* spp., respectively.

A clear correspondence was found between freshness state as defined by QI and the microbial counts. Specifically, the r of the QI with the TVC and *Pseudomonas* spp. for the Control fillets were 0.90 (*p*_r_ = 0.036) and 0.91 (*p*_r_= 0.030), respectively, whereas the corresponding values for the Treated fillets were 0.90 (*p*_r_ = 0.036) and 0.88 (*p*_r_ = 0.048).

### 3.2. Sensory Profile of Cooked Fillets

In total, 31 out of 37 evaluated sensory attributes varied significantly (*p* < 0.05) or showed a tendency (*p* < 0.1) according to the factorial study design (Table 3, Table 4, Table 5, Table 6 and Table S1). From the six attributes that did not vary significantly (*p* ≥ 0.1), two belonged to the odor modality (earthy and sulfuric), one to the appearance modality (wet-succulent) and three to the texture modality (firm, elastic, chewy) (Table S1). 

Six attributes varied only according to the treatment independently of the storage day; these concerned in-mouth (taste and flavor), aftertaste and mouthfeel attributes (Table 3). From those umami tastes, overall flavor intensity and buttery flavor, and mouthwatering sensation were perceived significantly higher in the Treated fillets than the Control ones. The opposite applied to salty aftertaste and mouth drying sensation.

Nine attributes varied only according to the storage day independently of the treatment; the majority of them belonged to the odor modality, whereas only two belonged to the flavor (Table 4). The pattern of variation depended on the attribute. Overall odor intensity, mussels, fermented, lactic acid, fishy off-odor and fermented flavor were perceived to increase significantly over the storage period. Overall odor intensity and mussel odor were perceived as significantly higher on D7 than on D1; fermented and lactic acid odor showed a significant gradual increase from D1 to D5 and thereafter D7; whereas fish off-odor and fermented flavor only increased significantly on D7. Marine odor and boiled potato odor and flavor exhibited the opposite pattern, showing a decrease along the storage period. For marine and boiled potato odor D7 was perceived as having a significantly lower intensity than D1, whereas for boiled potato flavor D7 showed a lower intensity than was perceived in both D1 and D5. Interestingly crustacean odor was perceived as having its peak intensity on D5 and a significantly lower intensity on D7, whereas D1 did not vary from either of the aforementioned days.

Ten attributes varied both according to the storage day and treatment factors, and can be divided mainly in two groups according to their pattern of variation with respect to both day and treatment (Table 5). Sweet taste and aftertaste, and grilled flavor were perceived significantly higher in the Treated fillets when compared to the Control, whereas their intensity decreased with storage, with D7 having significantly lower intensities than D1. On the contrary, bitter taste, lactic acid flavor, sour and bitter aftertaste and metallic mouthfeel were perceived higher in the untreated control fillets, whereas their intensity increased significantly on D7 when compared to D1 and D5; with the exception of lactic acid flavor that increased gradually from D1 to D5 and thereafter D7. Pasty texture variation pattern differed, since it was perceived higher in the Control fillets, while its intensity lowered during storage. 

Six attributes showed an interaction between treatment and storage day (Table 6). These concerned three appearance attributes, namely color intensity, compactness and flakiness. Color intensity increased during storage; this was perceived earlier within storage for the Treated fillets, which were first evaluated at D5 as having a significantly more intense color than D1, whereas this occurred on D7 for the Control fillets. Visual compactness decreased during storage only for the Treated fillets, whereas no significant variation was perceived for the Control ones. The significant variation that was perceived for flakiness amongst samples did not seem to follow a consistent pattern. Additionally, to the appearance attributes sour taste, metallic-mussels flavor and juicy texture also showed an interaction between treatment and storage. Sour taste and metallic flavor were perceived as having the lowest intensity at D1 for both the Control and Treated fillets. Their intensity increased during storage for the former but not the latter, since Treated fillets from D5 and D7 were not discriminated from D1 ones. The Treated fillets were perceived in general as having a juicier texture than the Control ones, with the exception of D5 control fillets, which exhibited similar intensities.

**Figure 3:**
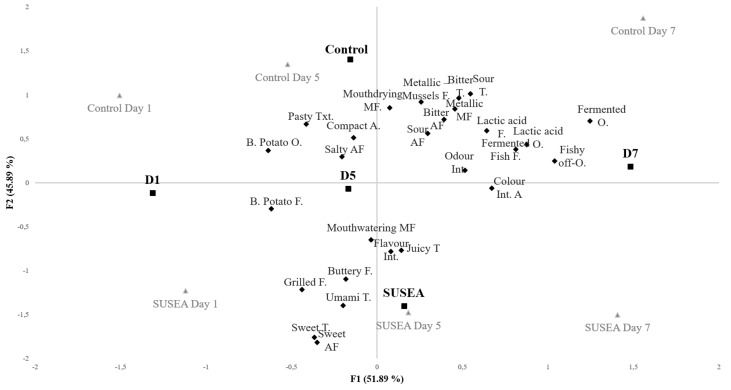
Principal Component Analysis (PCA) bi-plot of descriptive analyses (DA) data after Varimax rotation, including as observations the study design factors (■) and as variables (♦), descriptors with a *p* < 0.1 according to the factorial study. Study design factors, Treatment (Control (untreated) and SUSEA (treated) gilthead seabream fillets) and storage Day (D1, D5 and D7). DA samples are included as supplementary observations (▲). Total explained variance of factor 1, F1 and F2: 97.97%. O, A, T, F, Txt, AF and M stand for odor, appearance, taste, flavor, texture, aftertaste and mouthfeel, respectively.

According to the PCA of Figure 3, a slightly greater amount of attribute variance is explained by the storage (projected on factor 1, F1) when compared to the treatment (projected on F2) effects, accounting to 51.9% and 45.9%, respectively. When examining the variation of sensory quality with storage, D1 and D5 have more similar characteristics than D7. This is supported by the PCA bootstrap hulls of individual samples, which show that CD1 and CD5 (Control) as well as SD1 and SD5 (Treated) present overlapping sample areas (Figure S1B, Supplementary Material). Whereas the same applies for SD5 and SD7, CD7 bootstrap hull has no overlapping areas, indicating a discreet profile of developed characteristics that separates it from the rest of the samples.

**Figure 4:**
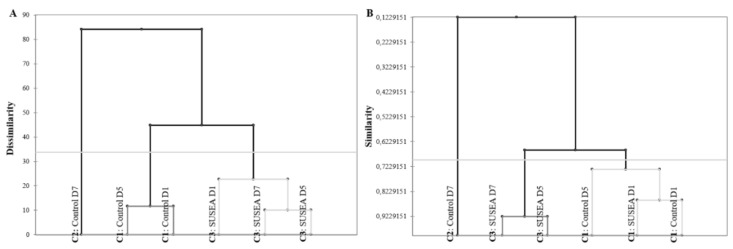
Agglomerative Hierarchical Clustering (AHC) dendrograms for (**A**) Dissimilarity and (**B**) Similarity samples (Control (untreated) and SUSEA (treated) gilthead seabream fillets) based on the descriptive analysis profiling data. C stands for cluster.

The variation of CD7’s sensory profile compared to the rest of the samples is clearly shown by both the similarity and the dissimilarity of AHC dendrograms indicating it is the least similar or the most dissimilar sample (Figure 4A,B). When examining the rest of the samples, D1 samples (CD1 and SD1) present the most common profiles, which are more closely related to CD5, and thereafter with a second group of samples consisting of SD5 and SD7.

## 4. Discussion

### 4.1. Processing Effects on the Fresh Fillets’ Quality and Shelf-Life Extension

The QI of both Control (untreated) and Treated (SUSEA) gilthead seabream fillets showed a linear increase with storage time [6]. The end of the observed shelf life for the Control fillets (7 days) coincided with the commercial use-by-date set on the package, as well as with previous works examining the shelf life of seabream fillets stored under similar conditions [26,31]. For osmotically dehydrated Treated fillets, a shelf-life extension of 29%, accounting to approximately two additional days, was achieved according to the QIM results. The ability of osmotic dehydration to extend the shelf life of fish, as associated to a decreased tissue water activity (*a_w_*) to values up to 0.95, is in accordance with previous species’ findings [15,25]. However, whereas it has been reported that dehydration treatments may result in the alteration of the raw fillets’ appearance and/or texture, according to the tetrad test, assessors evaluated the Control and Treated fillets as similar [15,36]. The lack of significant variation in the quality of raw fillets between the treatments as a function of dehydration could be partly attributed to the mild treatment applied, and it is considered crucial due to the role of appearance and texture in consumers’ acceptance and purchase of the final product [37,38,39]. 

In the present study, the initial TVC load of fish was relatively high and averaged 5.1 log CFU/g. However, the measured initial microbial loads were within the range reported in the literature for fresh gilthead seabream or European sea bass fillets [40,41,42,43,44]. *Pseudomonas* spp. dominated spoilage at the end of the storage period for both Control and Treated gilthead seabream fillets, which is in agreement with previous studies evaluating the shelf life of Mediterranean fish fillets during refrigerated storage at aerobic conditions [8,45]. Microbial growth was delayed after the treatment, which is in agreement with Neumeyer, et al. [46] who reported that a decrease of a_w_ to 0.95 will result in inhibition of *Pseudomonas* spp. growth.

Most of the references in the literature on the use of osmotic dehydration on fish evaluate the influence of different solutes (e.g., sucrose, corn starch syrup, NaCl) on the mass transfer into fish slices [27,47,48]. Tsironi et al. (2009) investigated the moisture loss and solid gain during osmotic dehydration of gilthead seabream fillets in a concentrated solution of maltodextrine and NaCl [49]. The treatments caused substantial water loss with higher solution concentrations showing the highest values of mass flow. Limited research has been reported about the application of osmotic dehydration of fish for shelf-life extension of fish products. The potential of osmotic dehydration using maltodextrine/NaCl solutions to delay the growth of spoilage bacteria and extend the shelf life of gilthead seabream fillets has been reported [16,25,49]. Microbial growth rates in osmotically treated fish fillets were significantly lower compared to untreated fillets stored at isothermal conditions within the range 0–15 °C. The decrease of the sensory scoring in the abovementioned studies had high correlation with microbial growth and total volatile basic nitrogen (TVB-N) production. *Pseudomonas* spp. growth was a good determinant for shelf-life evaluation and the acceptability limit was determined as 10^6^ cfu/g. According to the mathematical models developed by Tsironi and Taoukis (2017), the shelf life of gilthead seabream fillets during aerobic storage at 2 °C can be calculated as 8 days, while osmotic dehydration using alternative maltodextrine-based solutions may result up to 11 days at 2 °C, depending on the osmotic solution concentration and the selected osmotic media [26]. In the same study, storage at −3 °C resulted in almost 1 month shelf life of fish fillets at all the tested processing conditions. 

The applicability of osmotic dehydration for improving quality and extending shelf life of other fish species has been recently investigated, for example for yellowfin tuna [17], chub mackerel [50], European sea bass [18], eel [51] and tilapia [52,53]. The sensory profiling of the osmotically treated fish has not been yet reported in the literature.

### 4.2. Processing Effects on the Sensory Quality and Profile of Cooked Tissue 

Overall, the sensory attributes perceived in the cooked gilthead seabream tissue agree with previous literature describing the sensory profile of gilthead seabream [54,55,56,57,58]. 

Moreover, according to the modified Torry freshness scheme for gilthead sea bream, the overall variations observed with storage in the sensory profile of the cooked fillets agree with what is expected for the species. Indeed, attributes that were perceived to increase during storage, such as sour, bitter, lactic acid, fermented and fishy (off-) characteristics are more associated to “spoiled” tissue, whereas attributes such as sweet, boiled potato, crustacean (shellfish), marine (seaweed) are more characteristic of “fresh” gilthead seabream tissue [58,59]. With respect to metallic, whereas it has been mentioned as characteristic of fresh seabream tissue, its development can also indicate volatile lipid oxidation products in fish and fish oil enriched foods, explaining its increase during storage, especially when associated to fishy and mussel flavors [58,60]. This is in accordance with what should be expected in general regarding fish flavor according to Kawai and Sakaguchi [61], since fresh fish are characterized by mild and green/vegetative aromas, whereas more fishy-related attributes, associated to the oxidation of long chain fatty acids, develop during storage. 

When examining these variations in the perspective of the treatment effects on the sensory cooked quality of gilthead seabream fillets, it can be clearly seen that while the odor profile remained largely unaffected—mainly being dependent on the storage—the taste and flavor development of spoilage characteristics was hindered by the treatment. This includes attributes such as bitter taste, lactic acid flavor, sour-bitter aftertaste and metallic mouthfeel, which developed with storage in both treatments, however they were consistently perceived lower in the Treated fillets when compared to Control ones. Furthermore, for some of the spoilage related attributes (sour taste and metallic flavor), storage dependent development was only observed for the Control, whereas the Treated fillets retained their initial D1 intensities. Moreover, the treatment seemed to enhance and retain some fresh related characteristics, such as sweet taste and aftertaste, which while they decreased with storage, they were consistently perceived to be more intense in the Treated fillets. The sweet taste/aftertaste enhancement may be attributed to the glycerol and dextrose present in the osmotic solution. For grilled flavor, which shows the same pattern of variation as sweet taste, this enhancement can be associated once more to the treatment constituents, however this time indirectly, since glycerol can act as a flavor precursor in Maillard reactions [62]. Within these frames it seems that the sensory quality as evaluated by the QIM for the raw tissue is reflected in the cooked tissue, indicating a higher freshness quality perception for the Treated fillets, which can enhance consumer acceptance later in storage [23].

Beyond the spoilage—or freshness—dependent characteristics, certain sensory attributes were enhanced or suppressed only or mainly as a function of treatment. These included sensory attributes partly associated to Maillard reactions and/or savory corresponding sensations, including umami taste, overall flavor intensity, grilled flavor, buttery flavor and mouthwatering sensation [54,61,63,64,65]. Since the fillets were prepared under the same conditions (temperature, humidity), the higher perceived intensities of these attributes in the Treated fillets can be partly associated to the treatment constituents and the role of glycerol as a Maillard reaction flavor precursor, and salt as a flavor enhancer [62,66]

Whereas the QIM and tetrad discrimination test indicated no perceived variations in the raw quality of fillets between treatments early on within the shelf life, the cooked quality of the fillets varied as indicated by the PCA explained variance proportion of the treatment. These differences can be partly associated to the different degree of development of spoilage characteristics between the Control and Treated fillets, with samples early on in shelf life having more similar sensory profiles that in later stages. This applied especially to the Control fillet of D7 that developed a discreet profile as indicated by both AHC and PCA sample convex hulls. Considering the rest of the variations induced by the treatment, independently of storage related effects, these can only be evaluated in total as positive due to the sensory characteristics involved. Specifically, attributes as umami, butter and juiciness have been previously mentioned as hedonic drivers for fish and fish products, whereas the opposite applies for bitterness [67,68,69].

## 5. Conclusions and Directions for Future Research

The treatment achieved a shelf-life extension of 29%, as validated by both the Quality Index Method and microbial evaluation of gilthead seabream fillets. No deviations in the raw quality of the fillets were caused by the treatment in the beginning of shelf life as indicated by the results of the discrimination test. The cooked quality of the fillets profiled by sensory descriptive analysis varied with the treatment, exhibiting a slower development (lower intensities) for sensory attributes related to spoilage (bitter and sour taste and aftertaste, lactic acid and metallic—mussels flavor) for samples in the middle (D5) and end (D7) of shelf life. Moreover, a range of attributes were perceived as having higher perceived intensities in the Treated fillets when compared to the Control ones’, either independently of, or consistently during, shelf life. 

The results of the treatment are promising for the shelf-life extension of gilthead seabream fillets, however the authors argue that a more prolonged extension could have been achieved given a lower initial microbial load of the raw material (fillets) for processing. Furthermore, whereas the sensory attributes enhanced by the treatment in the cooked tissue can be evaluated in total as positive, further work examining consumers’ preferences for the Treated fillets and corresponding attributes versus the Control ones are needed for validation.

## Figures and Tables

**Table 1:** Quality Index Method (QIM) scheme developed for the evaluation of freshness of gilthead seabream fillets. The demerit points and their corresponding descriptions are included for each attribute parameter.

Modality	Attribute	Description	Demerit Points
Appearance	Color	White	0
		Greyish	1
		Yellowish	2
	Discoloration	Uniform color with no discolorations	0
		Slight discolorations	1
		Intense	2
	Brightness	Bright, lean, shiny, lucid	0
		Slightly bright, slightly porous	1
		Pale, porous, dull matt	2
Odor	Quality	Fresh seaweed ^1^	0
		Neutral ^2^	1
		Fishy ^3^	2
		Stale, off-odors ^4^	3
Texture	Firmness	Firm	0
		Slightly firm	1
		Soft	2
	Elasticity	Elastic	0
		Slightly elastic	1
		Non-elastic	2
Quality Index (QI)			0–13

^1^ Marine and fresh seaweed aroma; ^2^ Neutral and/or slight fresh melon/cucumber aroma; ^3^ Fresh melon/cucumber aroma with fishy notes; ^4^ Stale/rancid with fishy off-odors.

**Table 2:** Sensory attributes used in the final Descriptive Analyses (DA) evaluation, divided in modalities, along with their definitions and associated references used during the training sessions.

Attributes	Definition and Associated References
***Odor (O.)* ^1,2^**	
Intensity	Overall intensity of the odor of the sample as a whole
Marine	Associated to reference of raw shrimps, related to seawater/fresh seaweed
Crustacean	Associated to reference of cooked crab, cooked shrimp, cooked scallops
Boiled potato	Associated to reference of boiled potatoes and vegetable broth
Earthy	Associated to reference of raw dried mushroom
Mussels	Associated to reference of canned mussels and related to old seaweed
Fishy off	Associated to reference of anchovies, sardines and related to old fish
Fermented	Associated to reference of fish sauce
Lactic sour	Associated to reference of lactic acid
Sulfuric	Associated to reference of boiled eggs, boiled broccoli/cauliflower
***Appearance (A.)***	
Color intensity	The color intensity of the surface of the tissue ^3^
Compactness	The firmness appearance and tightness of the sample’s structure ^1^
Wet/Succulent	The amount of liquid perceived on the surface of the tissue ^1^
Flakiness	The amount of visible flakes associated to laminar structure ^4^
***Taste (T.)* ^1^**	
Sweet	Taste associated to sweet compounds like sucrose
Sour	Taste associated to sour compounds like citric acid
Bitter	Taste associated to bitter compounds like caffeine
Umami	Taste associated to umami compounds like monosodium glutamate -MSG-
***Flavor (F.)* ^1^**	
Intensity	Overall intensity of the flavor of the sample as a whole
Metallic—Mussels	Associated to reference of canned mussels and related to old seaweed
Boiled potato	Associated to reference of boiled potatoes and vegetable broth
Buttery	Associated to dairy products like butter or cream
Grilled	Associated grilled/cooked notes also associated to roasted chicken breast
Lactic acid	Associated to reference of lactic acid
Fermented	Associated to reference of fish sauce
***Texture (Txt.)* ^1^**	
Firmness	Required force to cut through the tissue (first bite), using the front teeth
Elastic	Degree to which the product tissue is bouncing back while chewing
Juicy	Amount of liquid released from the sample while chewing (first bites)
Chewy	Effort required to break down the tissue over several bites and form bolus
Pasty	Extent to which the tissue structure becomes a mass/paste over several bites.
***Aftertaste (AF.)* ^1,5^**	
Sweet	Remaining taste associated to sweet compounds like sucrose
Sour	Remaining taste associated to sour compounds like citric acid
Salty	Remaining taste associated to salty compounds like NaCl
Bitter	Remaining taste associated to bitter compounds like caffeine
***Mouthfeel (M.)* ^1,5^**	
Metallic	Remaining flavor associated to metallic notes like iron
Mouth drying	The degree to which the product creates dryness in the oral cavity
Mouthwatering	The degree to which the product creates salivation

^1^ Intensity scale anchors “None” to “Very high”; ^2^ Evaluation instructions: lift one side of the foil and evaluate most of the odor within the first few smells/sniffs; ^3^ Intensity scale anchors “Very bright white” to “Very pale yellow-white”; ^4^ Intensity scale anchors “Homogenous appearance” to “Visible flakes”. ^5^ Evaluated 20 s after swallowing.

**Table 3:** Mean intensities (150 mm scale) of attributes varying with Treatment (*p* < 0.1), according to the factorial 2-way ANOVA model (fixed factors: Treatment, Day, Interaction: Treatment*Day). Post hoc, Fischer LSD, lowercase letters indicate significant differences between treatments.

Attributes	*p*-Value	Control (Untreated)			Treated (SUSEA)		
	Treatment	Day 1	Day 5	Day 7	Day 1	Day 5	Day 7
***Taste (T.)***							
Umami	<0.001	5.73b	5.32b	4.19b	7.34a	7.59a	7.27a
***Flavor (F.)***							
Intensity	<0.001	7.47b	7.62b	7.75b	9.17a	8.77a	9.02a
Buttery	<0.001	5.33b	5.68b	4.05b	6.89a	6.79a	6.79a
***Aftertaste (AF.)***							
Salty	0.017	4.20a	4.30a	3.57a	3.63b	3.48b	3.22b
***Mouthfeel (M.)***							
Mouth drying	0.001	5.82a	5.94a	6.27a	4.49b	4.48b	4.77b
Mouthwatering	0.008	6.90b	6.92b	6.09b	7.71a	7.44a	8.07a

**Table 4:** Mean intensities (150 mm scale) of attributes varying with storage Day (*p* < 0.1), according to the factorial 2-way ANOVA model (fixed factors: Treatment, Day, Interaction: Treatment*Day). Post hoc, Fischer LSD, capital letters indicate significant differences between storage days.

Attributes	*p*-Value	Control (Untreated)			Treated (SUSEA)		
	Day	Day 1	Day 5	Day 7	Day 1	Day 5	Day 7
***Odor (O.)***							
Intensity	0.004	8.31B	8.30AB	9.71A	7.61B	9.20AB	9.28A
Marine	0.073	7.12A	6.27AB	5.72B	6.59A	6.8AB	5.81B
Crustacean	0.071	7.93AB	7.57A	5.82B	6.58AB	7.45A	6.69B
Mussels	0.090	4.81B	5.80AB	6.30A	5.51B	6.16AB	6.18A
Fishy off-	<0.001	3.82B	4.06B	6.78A	3.24B	4.89B	6.32A
Fermented	<0.001	3.18C	4.59B	7.02A	2.45C	3.91B	6.06A
Lactic sour	<0.001	3.77C	5.36B	6.60A	3.70C	4.53B	6.13A
***Flavor (F.)***							
Boiled potato	<0.001	6.10A	5.79A	4.10B	6.21A	6.02A	4.63B
Fermented	<0.001	2.06B	3.24B	5.32A	2.69B	2.67B	4.16A

**Table 5:** Mean intensities (150 mm scale) of attributes varying with Treatment and storage Day (*p* < 0.1), according to the factorial 2-way ANOVA model (fixed factors: Treatment, Day, Interaction: Treatment*Day). Post hoc, Fischer LSD, lowercase and capital letters indicate significant differences between treatments and storage days, respectively.

Attributes	*p*-Value		Control (Untreated)			Treated (SUSEA)		
	Treat.	Day	Day 1	Day 5	Day 7	Day 1	Day 5	Day 7
***Odor (O.)***								
Boiled potato	0.053	0.003	7.39A	6.21AB	4.69B	5.80A	5.11AB	4.91B
***Taste (T.)***								
Sweet	<0.001	0.009	6.83bA	5.89bAB	5.16bB	9.5aA	8.72aAB	8.33aB
Bitter	<0.001	0.002	4.16aB	5.02aB	6.19aA	3.14bB	3.51bB	4.25bA
***Flavor (F.)***								
Grilled	<0.001	0.002	6.32bA	6.35bA	4.32bB	8.15aA	7.42aA	7.21aB
Lactic acid	0.022	<0.001	3.46aC	4.72aB	5.86aA	3.16bC	3.75bB	4.72bA
***Texture (Txt.)***								
Pasty	<0.001	0.040	6.09aA	5.95aAB	5.56aB	5.57bA	4.32bAB	3.90bB
***Aftertaste (AF.)***								
Sweet	<0.001	0.004	5.88bA	4.16bB	3.90bB	8.15aA	7.56aB	7.27aB
Sour	0.025	0.096	3.39aB	4.21aAB	4.87aA	3.35bB	2.83bAB	3.74bA
Bitter	0.001	0.007	3.27aB	3.91aB	4.90aA	2.64bB	2.66bB	3.50bA
***Mouthfeel (M.)***								
Metallic	<0.001	0.003	5.67aB	6.09aB	7.66aA	4.78bB	5.12bB	5.68bA

**Table 6:** Mean intensities (150 mm scale) of attributes showing a significant (*p* < 0.05) interaction (Treatment*Day), according to the factorial 2-way ANOVA model (fixed factors: Treatment, Day and Interaction). Post hoc, Fischer LSD, lowercase letters indicate significant differences.

Attributes	*p*-Value	Control (Untreated)			Treated (SUSEA)		
	Interaction	Day 1	Day 5	Day 7	Day 1	Day 5	Day 7
***Appearance (A.)***							
Color intensity	0.011	5.52bc	5.8bc	7.88a	5.19c	8.02a	6.87ab
Compactness	0.027	9.03ab	9.73a	9.84a	9.56a	8.21b	8.10b
Flakiness	0.033	7.10a	5.27b	5.99ab	5.83ab	6.41ab	6.94a
***Taste (T.)***							
Sour	0.041	4.78c	5.62b	7.63a	4.14c	4.46bc	4.80bc
***Flavor (F.)***							
Metallic—Mussels	0.052	5.68bc	6.66ab	7.73a	5.29c	5.21c	5.11c
***Texture (Txt.)***							
Juicy	0.088	7.30c	8.89ab	8.01bc	9.68a	8.99ab	9.58a

## Data Availability

Not applicable.

## Supplementary Materials

The following supporting information can be downloaded at: https://www.mdpi.com/article/10.3390/foods11142017/s1, Figure S1: Principal Component Analysis (PCA) of descriptive analyses (DA) (A) bi-plot including as observations the DA samples (■) and as variables (♦), descriptors with a *p* < 0.1 according to the factorial study (B) Convex bootstrap hulls showing the discrimination amongst DA samples. Total explained variance of factor 1, F1 and F2: 90.68%. For DA samples, C and S stand for Control (untreated) and SUSEA (treated) fillets and D1, D5 and D7 for Day 1, 5 and 7, respectively. O, A, T, F, Txt, AF and MF stand for odor, appearance, taste, flavor, texture, aftertaste and mouthfeel, respectively. Table S1: Mean values and standard deviation (SD), between assessors, of attribute intensities measured 150 mm scale by descriptive analysis. Significance calculated by a factorial 2-way ANOVA model (fixed factors: Treatment, Day, Interaction: Treatment*Day) computed at 95% confidence level. Post hoc test Fischer LSD. Factors presenting a tendency (*p* < 0.1) are kept due to the high inherent variability between fillets.
