# Supplementary material for: Raw and Cooked Quality of Gilthead Seabream Fillets (Sparus aurata, L.) after Mild Processing via Osmotic Dehydration for Shelf Life Extension
Source: Foods. 2022 Jul 7;11(14):2017. doi: 10.3390/foods11142017 (PMC9318255; doi:10.3390/foods11142017)
Supplement: Supplementary file 1 [file foods-11-02017-s001.zip › foods-1779309-supplementary.pdf]

Supplementary materials

Raw and Cooked Quality of Gilthead Seabream Fillets (*Sparus aurata*, L.) after Mild Processing via Osmotic Dehydration for Shelf Life Extension

Niki Alexi [1,*], Konstantina Sfyra [1], Eugenia Basdeki [2,3], Evmorfia Athanasopoulou [2], Aikaterini Spanou [2], Marios Chryssolouris [3] and Theofania Tsironi [2]

[1] Food Quality Perception and Society Science Team, iSENSE Lab, Department of Food Science, Faculty of Technical Sciences, Aarhus University, Agro Food Park 48, 8200 Aarhus N, Denmark; ksfyra@food.au.dk

[2] Laboratory of Food Process Engineering, Department of Food Science and Human Nutrition, Agricultural University of Athens, Iera Odos 75, 11855 Athens, Greece; eugeniaba97@gmail.com (E.B.); efiatha@aua.gr (E.A.); sp.katerin@aua.gr (A.S.); ftsironi@aua.gr (T.T.)

[3] SuSea BV, High Tech Campus 1, 5656 AE Eindhoven, The Netherlands; mmc@susea.co

* Correspondence: niki.alexi@food.au.dk

Table S1. Mean values and standard deviation (SD), between assessors, of attribute intensities measured 150 mm scale by descriptive analysis. Significance calculated by a factorial 2-way ANOVA model (fixed factors: Treatment, Day, Interaction: Treatment*Day) computed at 95% confidence level. Post hoc test Fischer LSD. Factors presenting a tendency ($p < 0.1$) are kept due to the high inherent variability between fillets.

Attributes	Factorial 2-way ANOVA			Control (Untreated)						Treated (SUSEA)					
	**Treatment**	Day	Interaction	Day 1		Day 5		Day 7		Day 1		Day 5		Day 7	
				Mean	SD	Mean	SD	Mean	SD	Mean	SD	Mean	SD	Mean	SD
Odour (O.)															
Intensity	ns	0.004	ns	8.31B	1.77	8.30AB	1.23	9.71A	1.67	7.61B	1.64	9.20AB	1.38	9.28A	1.48
Marine	ns	0.073	ns	7.12A	3.3	6.27AB	2.2	5.72B	2.6	6.59A	2.8	6.8AB	2.7	5.81B	2.9
Crustacean	ns	0.071	ns	7.93AB	1.71	7.57A	1.34	5.82B	3.43	6.58AB	3.17	7.45A	1.94	6.69B	1.79
Boiled potato	0.053	0.003	ns	7.39A	2.64	6.21AB	3.21	4.69B	2.62	5.80A	2.33	5.11AB	2.88	4.91B	2.12
Earthy	ns	ns	ns	3.24	1.72	3.81	2.25	3.85	2.24	3.22	1.87	3.12	2.24	3.38	1.70
Mussels	ns	0.090	ns	4.81B	2.03	5.80AB	2.87	6.30A	2.45	5.51B	2.04	6.16AB	2.69	6.18A	2.63
Fishy off-	ns	< 0.001	ns	3.82B	1.81	4.06B	2.14	6.78A	3.28	3.24B	1.60	4.89B	2.92	6.32A	2.35
Fermented	ns	< 0.001	ns	3.18C	2.16	4.59B	2.52	7.02A	2.89	2.45C	1.39	3.91B	2.01	6.06A	2.34

Attributes	Factorial 2-way ANOVA			Control (Untreated)						Treated (SUSEA)					
	Treatment	Day	Interaction	Day 1		Day 5		Day 7		Day 1		Day 5		Day 7	
				Mean	SD	Mean	SD	Mean	SD	Mean	SD	Mean	SD	Mean	SD
Lactic sour	ns	< 0.001	ns	3.77C	1.52	5.36B	2.44	6.60A	2.58	3.70C	1.66	4.53B	2.29	6.13A	2.68
Sulphuric	ns	ns	ns	4.77	2.50	5.07	2.56	4.97	2.70	5.01	2.95	3.99	2.08	5.42	2.26
Appearance (A.)															
Colour intensity	ns	0.001	0.011	5.52bc	1.94	5.8bc	2.41	7.88a	1.52	5.19c	1.73	8.02a	1.68	6.87ab	3.45
Compactness	0.017	ns	0.027	9.03ab	2.25	9.73a	2.31	9.84a	1.48	9.56a	2.71	8.21b	3.01	8.10b	2.74
Wet/Succulent	ns	ns	ns	7.85	1.85	9.27	2.01	9.13	3.24	8.44	2.66	7.67	2.67	8.41	2.55
Flakiness	ns	ns	0.033	7.10a	2.78	5.27b	2.41	5.99ab	2.27	5.83ab	2.29	6.41ab	2.91	6.94a	2.50
Taste (T.)															
Sweet	< 0.001	0.009	ns	6.83bA	2.89	5.89bAB	2.45	5.16bB	2.03	9.5aA	2.93	8.72aAB	3.79	8.33aB	3.00
Sour	< 0.001	< 0.001	0.041	4.78c	3.90	5.62b	3.67	7.63a	3.65	4.14c	2.78	4.46bc	3.06	4.80bc	2.61
Bitter	< 0.001	0.002	ns	4.16aB	3.28	5.02aB	4.00	6.19aA	3.78	3.14bB	2.23	3.51bB	2.32	4.25bA	3.35
Umami	< 0.001	ns	ns	5.73b	2.71	5.32b	2.36	4.19b	2.11	7.34a	2.61	7.59a	2.86	7.27a	2.70
Flavour (F.)															
Intensity	< 0.001	ns	ns	7.47b	2.47	7.62b	2.23	7.75b	1.99	9.17a	2.52	8.77a	2.21	9.02a	2.37
Metallic-Mussels	< 0.001	ns	0.052	5.68bc	2.50	6.66ab	3.30	7.73a	2.55	5.29c	2.91	5.21c	2.12	5.11c	2.90
Boiled potato	ns	< 0.001	ns	6.10A	3.26	5.79A	3.31	4.10B	2.59	6.21A	2.29	6.02A	2.17	4.63B	1.85
Buttery	< 0.001	ns	ns	5.33b	2.58	5.68b	2.65	4.05b	2.59	6.89a	2.90	6.79a	3.10	6.79a	3.48
Grilled	< 0.001	0.002	0.099	6.32bA	2.79	6.35bA	2.84	4.32bB	2.60	8.15aA	3.12	7.42aA	3.37	7.21aB	3.42
Lactic acid	0.022	< 0.001	ns	3.46aC	2.57	4.72aB	3.67	5.86aA	2.41	3.16bC	2.35	3.75bB	2.81	4.72bA	3.09
Fermented	ns	< 0.001	ns	2.06B	1.56	3.24B	2.74	5.32A	2.85	2.69B	2.00	2.67B	2.22	4.16A	2.71
Texture (Txt.)															
Firmness	ns	ns	ns	7.35	2.19	8.10	3.34	6.99	3.06	8.13	3.12	7.14	3.97	7.05	3.05
Elastic	ns	ns	ns	7.78	2.64	8.10	3.59	8.17	3.27	7.83	3.76	8.41	3.79	8.50	2.72
Juicy	0.002	ns	0.088	7.30c	1.19	8.89ab	2.28	8.01bc	2.40	9.68a	2.07	8.99ab	2.79	9.58a	2.73
Chewy	ns	ns	ns	9.49	2.87	8,63	3.39	8.83	2.72	8.83	2.29	8.55	3.43	8.32	3.24
Pasty	< 0.001	0.040	ns	6.09aA	3.40	5.95aAB	3.37	5.56aB	3.93	5.57bA	2.95	4.32bAB	2.78	3.90bB	2.46
Aftertaste (AF.)															
Sweet	< 0.001	0.004	ns	5.88bA	2.88	4.16bB	2.40	3.90bB	1.98	8.15aA	3.34	7.56aB	3.70	7.27aB	3.02
Sour	0.025	0.096	ns	3.39aB	2.21	4.21aAB	2.69	4.87aA	2.36	3.35bB	2.88	2.83bAB	2.39	3.74bA	2.53
Salty	0.017	ns	ns	4.20a	3.97	4.30a	4.19	3.57a	4.01	3.63b	3.38	3.48b	3.59	3.22b	2.89

Attributes	Factorial 2-way ANOVA			Control (Untreated)						Treated (SUSEA)					
	**Treatment**	Day	Interaction	Day 1		Day 5		Day 7		Day 1		Day 5		Day 7	
				Mean	SD	Mean	SD	Mean	SD	Mean	SD	Mean	SD	Mean	SD
Bitter	0.001	0.007	ns	3.27aB	2.29	3.91aB	3.07	4.90aA	2.26	2.64bB	2.33	2.66bB	2.30	3.50bA	2.63
Mouthfeel (M.)															
Metallic	< 0.001	0.003	ns	5.67aB	3.18	6.09aB	3.22	7.66aA	2.79	4.78bB	2.77	5.12bB	3.17	5.68bA	3.72
Mouthdrying	0.001	ns	ns	5.82a	2.15	5.94a	2.65	6.27a	1.97	4.49b	2.28	4.48b	2.24	4.77b	1.99
Mouthwatering	0.008	ns	ns	6.90b	2.97	6.92b	2.59	6.09b	3.55	7.71a	2.02	7.44a	2.10	8.07a	3.34

Different letters denote statistically significant groupings for samples according to Post-hoc analysis performed by the Fischer LSD test. Lowercase and uppercase letters are used to indicate post hoc groupings according to Treatment and storage Day, respectively. When an interaction of factors is present, the post-hoc groupings reflect only the interaction effects and are indicated with lowercase letters.

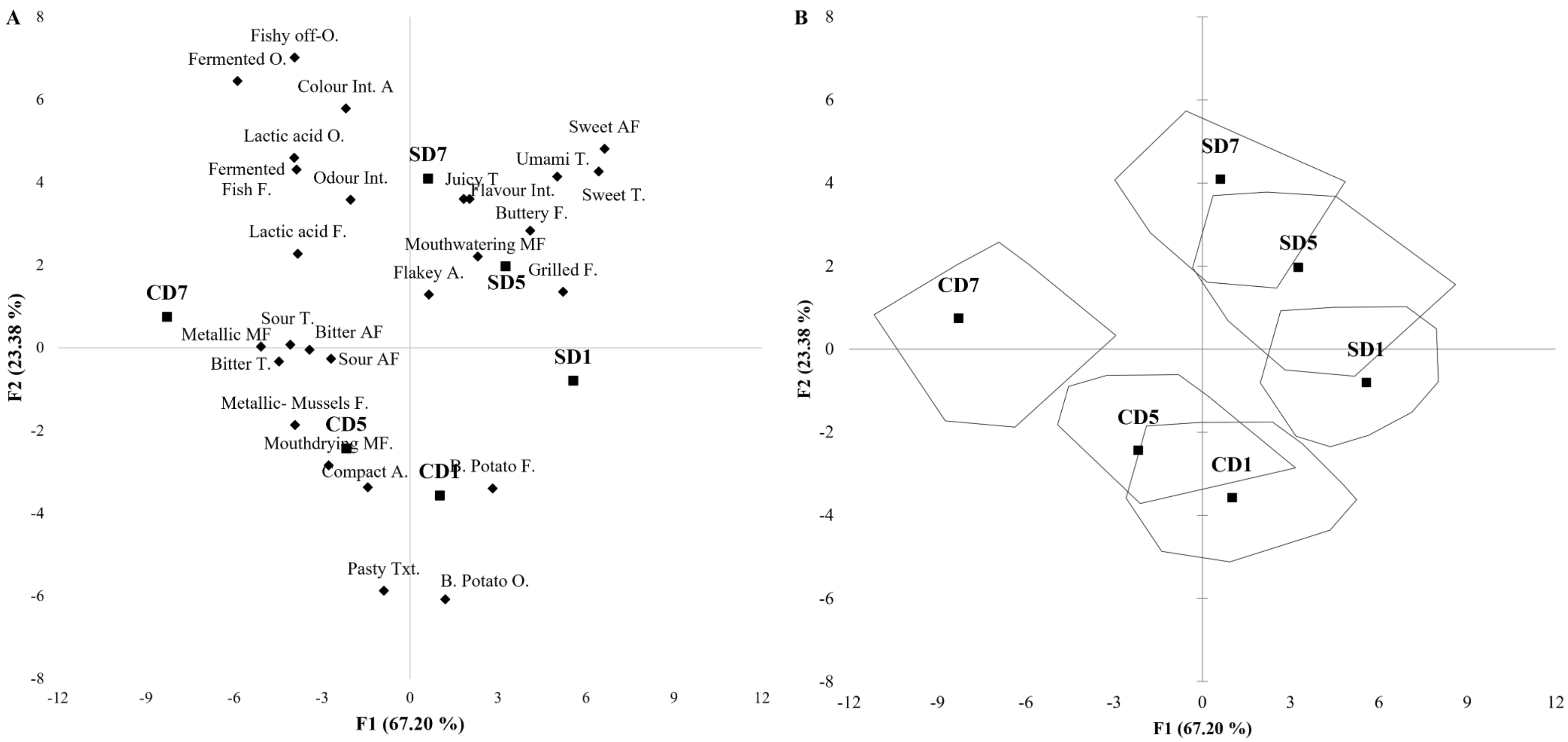

Figure S1. Principal Component Analysis (PCA) of descriptive analyses (DA) (A) bi-plot including as observations the DA samples (■) and as variables (◆), descriptors with a $p < 0.1$ according to the factorial study (B) Convex bootstrap hulls showing the discrimination amongst DA samples. Total explained variance of factor 1, F1 and F2: 90.68%. For DA samples, C and S stand for Control (untreated) and SUSEA (treated) fillets and D1, D5 and D7 for Day 1, 5 and 7, respectively. O, A, T, F, Txt, AF and MF stand for odor, appearance, taste, flavor, texture, aftertaste and mouthfeel, respectively.
